# Human, Nature, Dynamism: The Effects of Content and Movement Perception on Brain Activations during the Aesthetic Judgment of Representational Paintings

**DOI:** 10.3389/fnhum.2015.00705

**Published:** 2016-01-12

**Authors:** Cinzia Di Dio, Martina Ardizzi, Davide Massaro, Giuseppe Di Cesare, Gabriella Gilli, Antonella Marchetti, Vittorio Gallese

**Affiliations:** ^1^Department of Psychology, Università Cattolica del Sacro CuoreMilan, Italy; ^2^Department of Neuroscience, University of ParmaParma, Italy; ^3^Department of Art History and Archaeology, Columbia UniversityNew York, NY, USA

**Keywords:** experimental aesthetics, representational paintings, nature scenes, human figure, insula, embodiment

## Abstract

Movement perception and its role in aesthetic experience have been often studied, within empirical aesthetics, in relation to the human body. No such specificity has been defined in neuroimaging studies with respect to contents lacking a human form. The aim of this work was to explore, through functional magnetic imaging (*f* MRI), how perceived movement is processed during the aesthetic judgment of paintings using two types of content: human subjects and scenes of nature. Participants, untutored in the arts, were shown the stimuli and asked to make aesthetic judgments. Additionally, they were instructed to observe the paintings and to rate their perceived movement in separate blocks. Observation highlighted spontaneous processes associated with aesthetic experience, whereas movement judgment outlined activations specifically related to movement processing. The ratings recorded during aesthetic judgment revealed that nature scenes received higher scored than human content paintings. The imaging data showed similar activation, relative to baseline, for all stimuli in the three tasks, including activation of occipito-temporal areas, posterior parietal, and premotor cortices. Contrast analyses within aesthetic judgment task showed that human content activated, relative to nature, precuneus, fusiform gyrus, and posterior temporal areas, whose activation was prominent for dynamic human paintings. In contrast, nature scenes activated, relative to human stimuli, occipital and posterior parietal cortex/precuneus, involved in visuospatial exploration and pragmatic coding of movement, as well as central insula. Static nature paintings further activated, relative to dynamic nature stimuli, central and posterior insula. Besides insular activation, which was specific for aesthetic judgment, we found a large overlap in the activation pattern characterizing each stimulus dimension (content and dynamism) across observation, aesthetic judgment, and movement judgment tasks. These findings support the idea that the aesthetic evaluation of artworks depicting both human subjects and nature scenes involves a motor component, and that the associated neural processes occur quite spontaneously in the viewer. Furthermore, considering the functional roles of posterior and central insula, we suggest that nature paintings may evoke aesthetic processes requiring an additional proprioceptive and sensori-motor component implemented by “motor accessibility” to the represented scenario, which is needed to judge the aesthetic value of the observed painting.

## Introduction

The human capacity to experience the beauty of things is particularly evident in the creation and appreciation of works of art. Experiencing the aesthetics of artworks is a very intriguing and controversial subject dealt with by philosophers and, in comparatively recent years, by psychologists and neuroscientists. In the various studies investigating the processes involved in such a capacity, different levels of processing have been evaluated and discussed (see, for example, Chatterjee, 2003; Leder et al., 2004; Reber et al., 2004; Jacobsen et al., 2006; Cupchik et al., 2009; Locher et al., 2010). Chatterjee (2003) makes one of the first formal claims for the potential contribution of neuroscience to the study of aesthetics. In his review, he suggests a framework, adapted from visual cognitive neuroscience, from which hypotheses about visual neuroaesthetics can be tested. One very influential model in the theoretical definition of the various elements that may contribute to the aesthetic experience is Leder et al.'s (Leder et al., 2004; Leder, 2013) stage model. This “information-processing flow model” identifies a sequence of processing stages that represent different components of aesthetic processing. These components have recently been related to specific brain areas based on findings from empirical aesthetics (Leder et al., 2015). The present work intends to contribute to the explanatory power of such models by providing evidence from neuroimaging on the neural underpinnings associated with two fundamental factors—content and dynamism—that have been shown to influence aesthetic processing of artworks. By content we specifically refer to “what” is represented in the artwork (i.e., a nature scene vs. a human being) and by dynamism we refer to the perceived movement within the represented content.

Under a specific aesthetic condition or context (Cupchik and Laszlo, 1992; Leder et al., 2004; Di Dio et al., 2007; see also Höfel and Jacobsen, 2007; Cupchik et al., 2009; Kirk, 2008; see also Brieber et al., 2014), most models recognize at least three basic stages of aesthetic processing: a perceptual, a cognitive and an emotional stage. These stages are generally described compartmentally, although they are shown to affect each other continuously in the processing of the aesthetic experience even at the initial stages of object processing. Motion perception represents a meaningful example of such interactions. The analysis of motion involves both low-level processing of features like orientation and color, and high-level processing associated with factors such as the represented content. With respect to low-level processing, Gori et al. (2008) showed that, in Western visual art, motion perception in garments is evoked by the adoption of visual features such as orientation, curvature, and convergence of lines. Massaro et al. (2012) showed that color potentiates the aesthetic effect of paintings representing nature scenes judged as dynamic, possibly by enriching the image with perceptual details (i.e., increased image complexity, see Arnheim, 1992; Zellner et al., 2010). In neuronal terms, using rather complex stimuli (Thakral et al., 2012) measured brain activity within the visual sensitive motion area M+ while participants viewed van Gogh paintings classified as either pleasant or unpleasant and as more or less dynamic. The results confirmed that M+ is involved in processing implied motion. Viewing paintings produced a very realistic perceptual experience in which approximately half of the elements in the paintings appeared to be in motion. However, motion processing in M+ was not associated with aesthetic perception. By focusing on specific regions of interest involved in low-level motion processing, the authors did not account for the contribution of regions involved in high-level visuo-motor analysis, such as prefrontal and parietal areas. This is most critical considering that participants were presented with quite rich pictorial representations. In this respect, a recent TMS work by Cattaneo et al. (2015) showed that visual area V5 is sensitive to motion when attending to abstract and representational paintings, but only to the aesthetics of the abstract stimuli (and not the representational ones), in which attention is possibly focused on low-level visual features (e.g., Cupchik et al., 2009; Nadal, 2013).

Different aspects of movement are processed in distinct cortical brain areas, including, besides the primary visual cortex, the parietal and temporal cortices (Perrett et al., 1989; Allison et al., 2000; Pelphrey et al., 2004; Thompson et al., 2005), as well as frontal regions, including primary and premotor areas (Gallese et al., 1996; Rizzolatti et al., 1996; Binkofski et al., 1999; for a review, see Rizzolatti and Sinigaglia, 2010). The involvement of motor-related structures when viewing artistic representations was shown by Battaglia et al. (2011) in a study employing transcranial magnetic stimulation (TMS). They analyzed corticomotor excitability during the observation of a painting portraying an action vs. observation of a painting showing the same muscles at rest. Observation of the painting with implied motion produced increased cortical excitability, offering a motor correlate of the relationship between the artistic quality of a work and the perception of implied movement within it.

The majority of the studies specifically investigating movement perception and aesthetic processing benefit from a particular content: the representation of the human body. In this respect, a variety of neuroimaging studies have shown that the aesthetics of the human form engages higher visual areas (e.g., the extrastriate body area—EBA; superior temporal sulcus—STS; medial temporal—MT-complex), as well as areas known to be part of the motor and emotion-mirror mechanisms (for reviews see Peelen and Downing, 2005, 2007; Di Dio and Gallese, 2009). Investigating the brain correlates associated with the aesthetic experience for artworks, Di Dio et al. (2007) carried out functional MRI (fMRI) while participants observed and explicitly evaluated the aesthetics of images portraying Classical and Renaissance sculptures representing the human body or images of real human bodies. Among the visual activations, signal increase was found for both stimulus categories relative to baseline in the lateral occipital cortex (LOC) and the inferior temporal lobe (shape-sensitive areas), as well as in the medial temporal/medial superior temporal (MT/MST) complex. The MT/MST complex is shown to be involved in the analysis of motion (e.g., Watson et al., 1993) as well as by the vision of static images implying motion (Kourtzi and Kanwisher, 2000). Most noteworthy was the activation of the inferior parietal lobule and of the premotor cortex. These areas are known to become active during the observation of actions performed by others (see Rizzolatti and Sinigaglia, 2010) and it is likely that their activation was dependent on the intrinsic dynamic properties of the human bodies and on the sense of action that they evoked in the observer. This interpretation is in line with results from Proverbio et al. (2009), who presented participants with static pictures of women and men engaged in simple dynamic and almost static actions while event related potentials (ERPs) were recorded. Observation of static photographs of human actions with implied motion produced an increase in cortical activation, much greater for dynamic than less dynamic actions. The direct contrast between dynamic and static images highlighted enhanced activation for the dynamic stimuli in various areas, including V5/MT, EBA, STS and premotor and motor areas, suggesting that observation of static photographs of human actions with implied motion is able to activate structures involved in visuo-motor processing. In a TMS study, Calvo-Merino et al. (2010) explored the effect of the aesthetics of the human body on the activations of ventral premotor cortex and EBA. They applied repetitive TMS (rTMS) to disrupt aesthetic processing while healthy volunteers made aesthetic preference judgments between pairs of dance postures and non-body stimuli. rTMS over EBA, a posterior temporal section critical for the analysis of complex forms, including the body parts, resulted in a reduced aesthetic evaluation of body stimuli (but not of non-body stimuli).

From these studies, the relationship between the neural coding of a human body and motor processing is evident. Within artworks that lack a human form, however, this relationship remains fairly unexplored and, in our view, studying the role of movement perception in aesthetic processing for different art contents would be more comprehensive. Accordingly, the present work explored the role of movement perception in aesthetic processing by varying the content of the presented paintings and, more specifically, by comparing activations observed for representational paintings portraying a human figure as opposed to nature scenes lacking a human form.

Evidence that movement perception in artworks is somehow related to the depicted content was found in Massaro et al. (2012). In their eye-tracking study, the authors studied the relationship between content and perceived movement in representational paintings, where the represented content could be either a nature scene or a scene that included a human subject. The stimuli were categorized as dynamic or static and presented to the participants in a color and color-desaturated version. Interaction analyses showed that the absence of information about color did not affect the aesthetic evaluation of stimuli portraying a dynamic human subject. In line with the neuroscientific evidence described above, a human body might in fact imply an intrinsic and natural dynamism, evoking motor resonance in its beholder through attention to features that describe actions and emotions, such as the limbs and the face (for a theoretical review, see Freedberg and Gallese, 2007). In contrast, when rating the aesthetics of nature content paintings, aesthetic evaluation of dynamic images dropped appreciably in the absence of low-level sensory information (i.e., color), suggesting that dynamism of nature scenes involves perceptual analysis.

From neuroimaging, it is known that the observation of nature scenes in paintings activates structures involved in self-referential experiences, such as cuneus, precuneus, and medial temporal areas, including the lingual gyrus (Mizokami et al., 2014; for a review, see Vartanian and Skov, 2014) and the parahippocampus (Yue et al., 2007). Presenting participants with images depicting a variety of scenes (natural vistas, city streets, rooms, etc.), Yue et al. (2007) showed that higher activity in the parahippocampal place area was associated with increased scene preference (see also Lewis et al., 1981; Biederman and Vessel, 2006). In Kawabata and Zeki (2004), still life produced the greatest change at V3 and landscapes at the parahippocampal place area. Silveira et al. (2012) compared naturalistic vs. surrealistic paintings using fMRI and found, for paintings representing nature scenes, a significantly higher activation in the precuneus, medial occipital cortex bilaterally and in right-middle temporal areas. Directly comparing attractiveness of face and place images, Pegors et al. (2015) showed that, behaviorally, there was no difference in preference ascription between the two stimulus-categories, although there was a trend for preference for places. The neuroimaging (*f* MRI) data showed that, within ventromedial prefrontal cortex (vmPFC), along with category-specific activations, there was overlapping activation in response to attractive images, which was independent of stimulus category, suggesting that the positive reward properties of these two types of stimuli undergo similar processing.

Observation of nature scenes in paintings is further shown to activate the posterior parietal cortex (Kawabata and Zeki, 2004; Cela-Conde et al., 2009; Cupchik et al., 2009), a region involved in visuo-spatial coding as well as motor mapping (for a review, see Fogassi and Luppino, 2005). Investigating gender-related similarities and differences in the neural correlates of beauty using magnetoencephalography (MEG), Cela-Conde et al. (2009) presented participants with images of unfamiliar paintings and “natural” photographs depicting different objects and landscapes, urban and rural. The participants were required to rate each stimulus as beautiful or not. The results showed enhanced activation for “judged-beautiful vs. judged-ugly” stimuli in several parietal foci, bilaterally for women and mainly in the right hemisphere for men, with a latency of 300 ms after stimulus offset. Early activation of motor and somatosensory areas suggested that the aesthetic processing of artworks may involve increased spatial, cognitive, somatosensory, and motor (planning and execution) activity. “Viewers would “navigate,” so to speak, through the space offered by the beautiful image” (p. 3848). This interpretation gives rise to the idea that the observation of a nature scene not only involves a fine analysis of the visual features characterizing the artwork, but also that the motor system may be actively engaged by the depicted representations. What lacks in previous work is a clear connection between movement perception and this motor processing.

In the present fMRI study we investigated the effect of movement perception on brain activations when participants viewed representational paintings depicting either nature scenes or human figures. The stimuli were categorized a priori as static or dynamic by independent evaluators and were presented in three tasks: observation, aesthetic judgment and movement judgment. Observation task was introduced to outline spontaneous, task-unrelated, processes associated with aesthetic experience, whereas movement judgment task aimed at outlining activations specifically associated with movement processing, so as to better describe the nature of the activations found during aesthetic judgment. The particular aim we had with the present work was to contribute toward an integrated vision of aesthetic processing, offering new insights from neuroimaging with respect to the relationship between movement perception and the represented content.

### Predictions for the study

In general, we predicted specific activation of areas involved in the processing of each category of stimulus as a function of content (nature, human). With respect to paintings containing a human figure, we anticipated the involvement of the motor mirror mechanism, particularly for paintings categorized as dynamic (action description). With respect to nature content paintings, in line with other studies, we predicted the involvement of primary visual areas and deep temporal areas (e.g., hippocampus and lingual gyrus) involved in fine visual descriptions of the stimuli. Additionally, we hypothesized enhanced activation of posterior parietal cortex, involved in visuo-spatial and motor processing, in response to stimuli judged as dynamic compared to static.

## Materials and methods

### Participants

Nineteen healthy right-handed undergraduate university volunteers, without formal knowledge in art (11 females, 8 males; mean age = 27.16 years, SD = 3.47, age range = 23–37 years; mean schooling = 17.58 years, SD = 0.69) participated in the study. All participants had normal or corrected-to-normal visual acuity. They gave their written informed consent to the experimental procedure, which was approved by the Local Ethics Committee (Parma).

### Experimental design

The experimental design of the study is a 2 × 2 factorial with two levels of content (*nature, human*) and two levels of dynamism (*dynamic, static*). The stimuli were presented to the participants in three experimental tasks: observation (OBS), aesthetic judgment (AJ), and movement judgment (MJ). With respect of our previous eye-tracking studies (Massaro et al., 2012; Savazzi et al., 2014), here we introduced an observation task to outline the spontaneous activation of areas involved in processing the aesthetics of the stimuli. The study was carried out in one experimental session.

### Stimuli

Twenty-four digital images of paintings were chosen from the database of a previous work (Massaro et al., 2012; see also Savazzi et al., 2014), in which unfamiliar representational paintings were selected from an initial pool of 100 stimuli (for details on stimulus selection, see Supplementary Material in Massaro et al., 2012). The stimuli included artworks representing human full-figures and outdoor nature landscapes, which were further categorized according to the level of perceived movement, as judged by three independent evaluators, and subsequently confirmed by the judgments expressed by the participants of the present study during movement judgment task. A full description of the stimuli is in Massaro et al. (2012; Supplementary Material).

**Figure 1:**
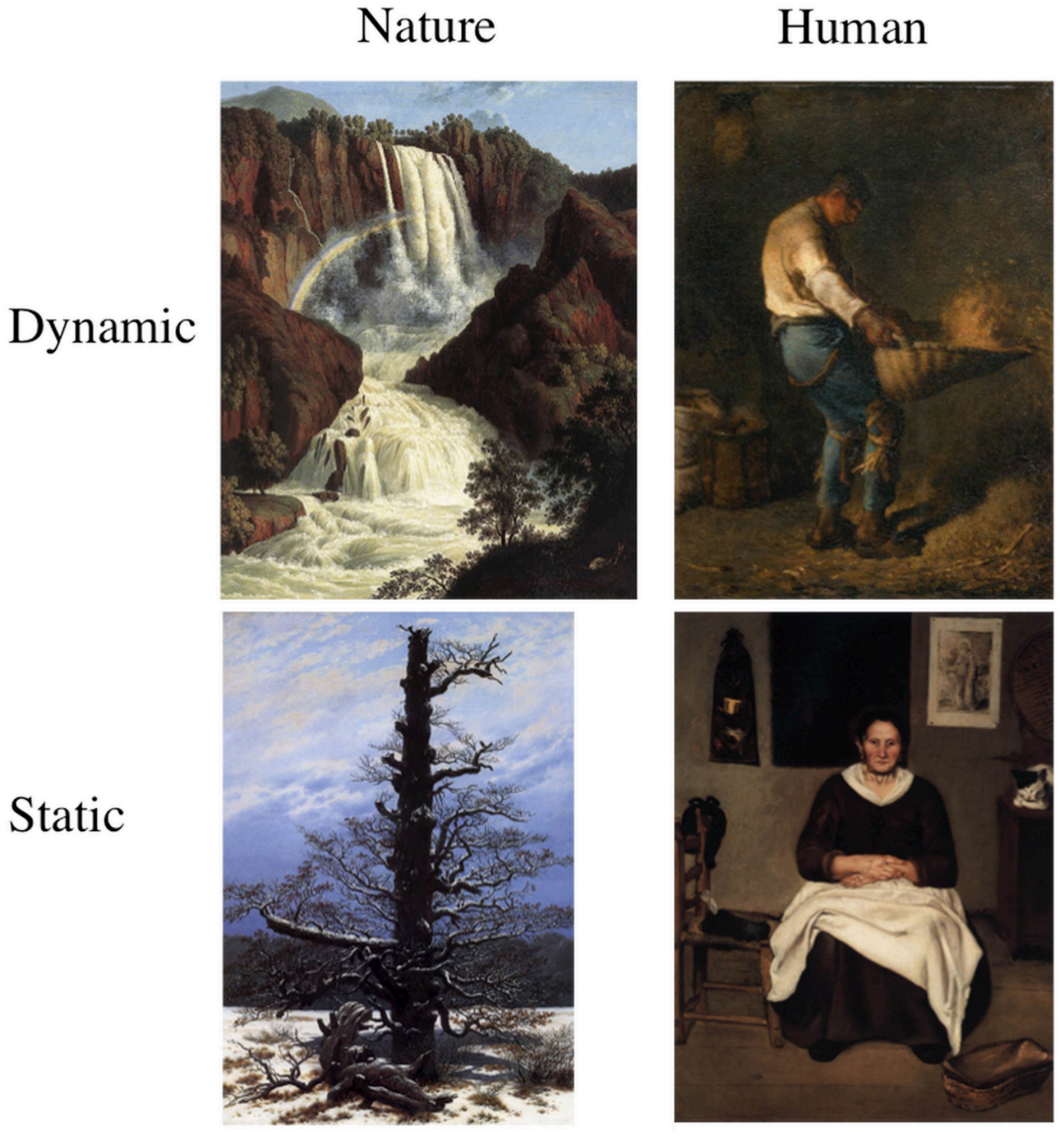
**Example of stimuli used in this study presenting, starting from left to right, a nature and a human content painting categorized as dynamic (top figures) and a nature and human content painting categorized as static (bottom figures)**.

From this initial pool of stimuli, 6 human dynamic (HD), 6 human static (HS), 6 nature dynamic (ND), and 6 nature static (NS) images were selected, totalling 24 stimuli (an example of the stimuli is shown in Figure 1). The human content paintings all contained only one human figure embedded in a context from which it emerged as the main element. Five images represented a male subject whereas seven portrayed a female figure. With respect to nature content stimuli, the static paintings mostly represented landscape scenarios (e.g., valleys), whereas the dynamic images included portrayals of water scenarios (seas and falls). A detailed description of the 24 stimuli used in this study, including category, title, artist, year of production, collection, content description is reported in Supplementary Table S1A.

Immediately after scanning, the participants were required to respond in a yes/no forced choice task whether they had seen the stimuli before the study. All participants reported that they had not seen any of the paintings before (100% unfamiliarity rating). Additionally, *post-hoc* ratings for familiarity measured on a seven point likert scale (0–6; not familiar at all—very familiar) collected on a different sample of subjects (*N* = 20) confirmed that the stimuli are generally not known by viewers with no formal knowledge in art (response range: 0.05–1.5; mean = 0.75, S.E. = 0.08). Different perceptual dimensions of the stimuli, including complexity, arousal, emotional valence and content valence were also assessed. The scores for complexity showed no perceptual differences between any of the stimulus categories, which were generally perceived as not very complex (*P* > 0.05; mean = 2.5, S.E. = 0.3). A cognitive and emotional assessment of the stimuli further showed that nature content stimuli scored higher than human content paintings on content valence, emotional valence and arousal (*P* < 0.05). Supplementary Table S1B reports the ratings recorded for complexity, arousal, content valence, and emotional valence.

During scanning, the aspect ratio of the paintings was preserved adjusting the image size to a maximum dimension of 800 × 600 pixels.

### Paradigm and task

During the fMRI acquisition, participants lay in the scanner in a dimly lit environment. The stimuli were viewed via digital visors (VisuaSTIM) with a 500,000 px × 0.25 square inch resolution and horizontal eye field of 30°. The digital transmission of the signal to the scanner was via optic fiber. The software E-Prime 2 Professional (Psychology Software Tools, Inc., Pittsburgh, USA, http://www.pstnet.com) was used both for stimuli presentation and the recording of the participants' answers.

The stimuli were presented under three tasks: observation (OBS), aesthetic judgment (AJ), and movement judgment (MJ). The order of the tasks was maintained fixed across participants so as not to impair brain response with preceding tasks. In particular, observation task was used to outline processes observed in AJ that can be also evoked spontaneously in the viewer. Movement judgment task, on the other hand, aimed at outlining activations found in AJ that are also involved in explicit movement judgment. By keeping AJ first, we aimed at avoiding the influence of prior evaluation of movement on explicit aesthetic assessment.

At the beginning of each run/task, a 20 s visual instruction informed the volunteers about the upcoming task. In order to set the proper aesthetic “mind” set (see, e.g., Cupchik and Laszlo, 1992; Leder et al., 2004; Di Dio et al., 2007; Höfel and Jacobsen, 2007), during OBS the participants were instructed to pretend to be in an art gallery, relax, and observe the images in their entirety. All tasks (OBS, AJ, MJ) required a motor response from the participants. During OBS, the subjects had to press a key at random whenever a red circle appeared on the screen, trying to alternatively select all keys. During AJ task, they were instructed to indicate, on the appearance of a question mark, how beautiful was the painting they had just seen, whereas during MJ task they had to indicate to what extent the painting they had just seen expressed movement. Judgments were recorded on a scale ranging from 1 to 4, where 1 represented the lowest score (not beautiful at all/no movement at all) and 4 the highest score (very beautiful/very much movement). Each finger corresponded to one specific response: the thumb, index, medium, and ring finger produced responses 1, 2, 3, 4 respectively. Since increasing numbering corresponded to increasing perceptual evaluations, the response order was not counterbalanced. Due to experimental time constraints, during AJ and MJ tasks the participants were required to respond only on one third of the trials (catch trials). Since each of the six images representing a specific stimulus category (HD, HS, ND, NS) was repeated 5 times, totalling 30 repetitions for each category, this means that we recorded 2 responses for each stimulus. On average, about 88% of the stimuli were rated congruently between repetitions (HD = 82.46%; HS = 94.74%; ND = 88.6%; NS = 85.09%).

The stimuli were presented for 2.75 s, preceded by a 250 s fixation cross and followed by a jittered interval ranging 3–12 s. For AJ and MJ tasks, on the catch trials, the stimulus was followed by a 2 s question mark prompting the appropriate response as per task request. Responses were always followed by a jittered ITI ranging 1–7 s to reduce the effect of finger movement on successive trials. Additionally, the catch trials were presented in a random fashion, so that the subjects were unaware of the exact response timing and hence were prompted to always evaluate the stimuli. The behavioral results for AJ and MJ tasks are shown in Figure 1. The AJ and MJ scores produced for each one painting are reported in Supplementary Table S1B. The complete dataset with the participants' responses can be found in Supplementary Material.

### fMRI data acquisition

Anatomical T1-weighted and functional T2^*^-weighted MR images were acquired with a 3 Tesla General Electrics scanner equipped with an 8-channel receiver head-coil. Functional images were acquired using a T2^*^-weighted gradient-echo, echo-planar (EPI) pulse sequence (acceleration factor asset 2, 40 sequential transverse slices covering the whole brain, with a TR time of 3000 ms, TE = 30 ms, flip-angle = 90 degrees, FOV = 205 × 205 mm2, inter-slice gap = 0.5 mm, slice thickness = 3 mm, in-plane resolution 2.5 × 2.5 × 2.5 mm^3^). At the beginning of the functional runs/sessions a T1-weighted anatomical scan (acceleration factor arc 2, 156 sagittal slices, matrix 256 × 256, isotropic resolution 1 × 1 × 1 mm3, TI = 450 ms, TR = 8100 ms, TE = 3.2 ms, flip angle 12°) was acquired for each participant.

#### Statistical analysis

Data analysis was performed with SPM8 (Statistical Parametric Mapping software; The Wellcome Department of Imaging Neuroscience, London, UK; http://www.fil.ion.ucl.ac.uk) running on MATLAB R2009b (The Mathworks, Inc., Natick, MA). The first four volumes of each run were discarded to allow for T1 equilibration effects. For each participant, all volumes were spatially realigned to the first volume of the first session and un-warped to correct for between-scan motion, and a mean image from the realigned volumes was created. T1 weighted images were realigned to create a mean image and then segmented into gray, white and cerebrospinal fluid and spatially normalized to the Montreal Neurological Institute (MNI). Thereby derived spatial transformation by T1 normalization was applied to the realigned EPIs volumes, which after normalization were re-sampled in 2 × 2 × 2 mm^3^ voxels using trilinear interpolation in space. All functional volumes were then spatially smoothed with a 6-mm full-width half-maximum isotropic Gaussian kernel for the group analysis.

Data were analyzed using a random-effects model (Friston et al., 1999), implemented in a two-level procedure. In the first level, single-subject fMRI responses were modeled in a General Linear Model (GLM) by a design-matrix comprising the onsets and durations of each event for each functional run/task (HD, HS, ND, NS, Response). The presentation of the stimuli for each trial-condition was modeled as one mini-epoch lasting 2.75 s, whereas the motor response as one single event lasting 0 s. In the second level analysis (group-analysis), corresponding contrast images from the first level for each participant were entered into flexible ANOVAs with sphericity-correction for repeated measures (Friston et al., 2002) independently for each task (*OBS, AJ, MJ*). These models considered the pattern of activation obtained within each tasks as a function of stimulus-content (Nature, Human) and stimulus-dynamism (dynamic, static) vs. implicit baseline (fixation cross), as well as activations resulting from the direct contrast between factors.

All results were thresholded at *p* < 0.05 family wise error (FWE) corrected at the cluster level (cluster size estimated with a voxel-level threshold of p-uncorrected = 0.001). The location of foci of activation is presented in the stereotaxic space of the MNI coordinate system.

## Results

### Response to the stimuli during AJ and MJ tasks—behavioral analysis

#### AJ scores

**Figure 2:**
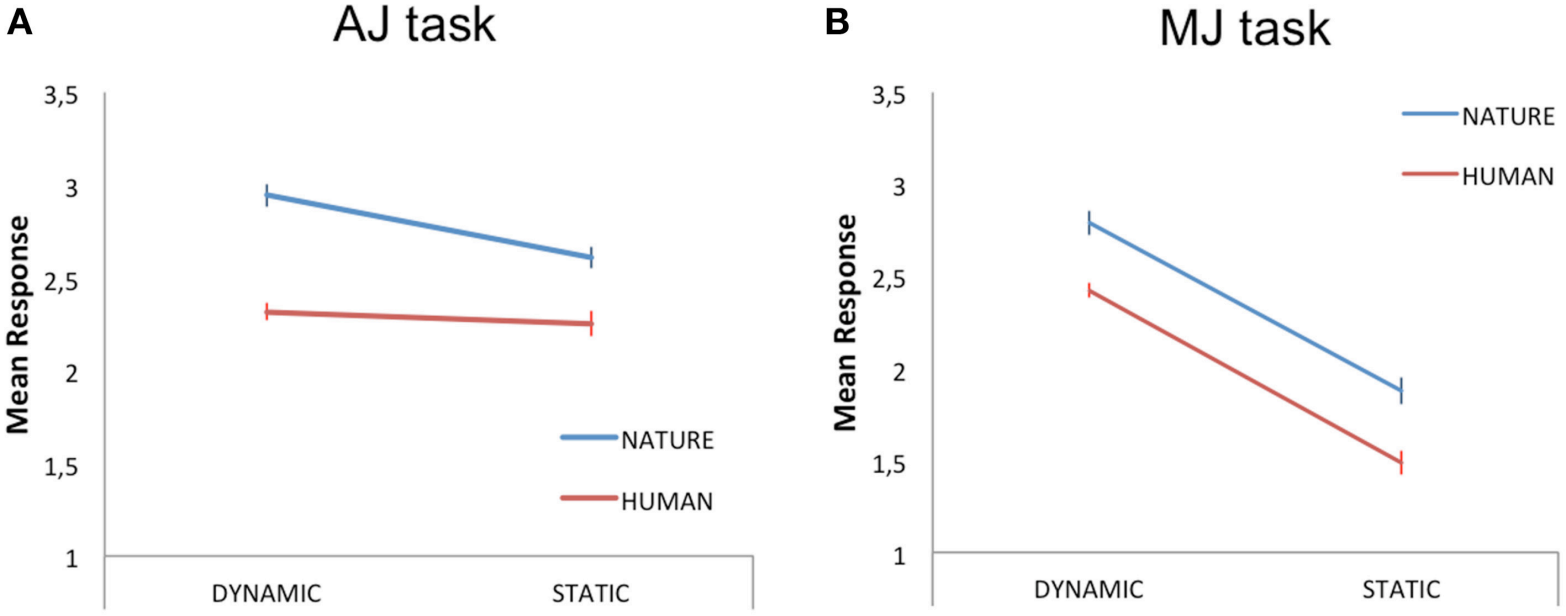
**Mean judgment scores for the dynamic and static human (red line) and nature (blue line) content paintings during (A) aesthetic judgment—AJ—and (B) movement judgment—MJ—tasks**. The bars represent the standard error of the mean.

Within this analysis, we averaged the participants' responses to each stimulus category (HD, HS, ND, NS) and carried out a 2 × 2 repeated measures GLM analysis, with two levels of stimulus-content (human, nature) and two levels of stimulus-dynamism (dynamic, static) as independent variables (IVs) and aesthetic judgment as the dependent variable (DV). The results revealed a main effect of content [N > H; *F*_(1, 18)_ = 6.80, *p* < 0.05, partial-η^2^ = 0.27, δ = 0.7] and a main effect of dynamism [D > S; *F*_(1, 18)_ = 5.16, *p* < 0.05, partial-η^2^ = 0.22, δ = 0.58], with nature and dynamic stimuli receiving higher aesthetic scores than human and static paintings, respectively (Figure 2A).

#### MJ scores

A 2 × 2 repeated measures GLM analysis, with two levels of stimulus-content (human, nature) and two levels of stimulus-dynamism (dynamic, static) as IVs and movement judgment as DV, revealed a main effect of content [N > H; *F*_(1, 18)_ = 5.98, *p* < 0.05, partial-η^2^ = 0.25, δ = 0.64] and a main effect of dynamism [D > S; *F*_(1, 18)_ = 52, *p* < 0.001, partial-η^2^ = 0.74, δ = 1; Figure 2B].

#### Correlation AJ—MJ tasks

The correlation analyses carried out between responses recorded during AJ and MJ tasks as a function of stimulus content and perceived dynamism showed a significant positive correlation between the aesthetic and movement evaluations of human content paintings [Pearson's *r*_(19)_ = 0.66, *P* < 0.01] but not of nature stimuli [Pearson's *r*_(19)_ = 0.43, *P* = 0.06].

### fMRI analysis

#### Global activations

**Figure 3:**
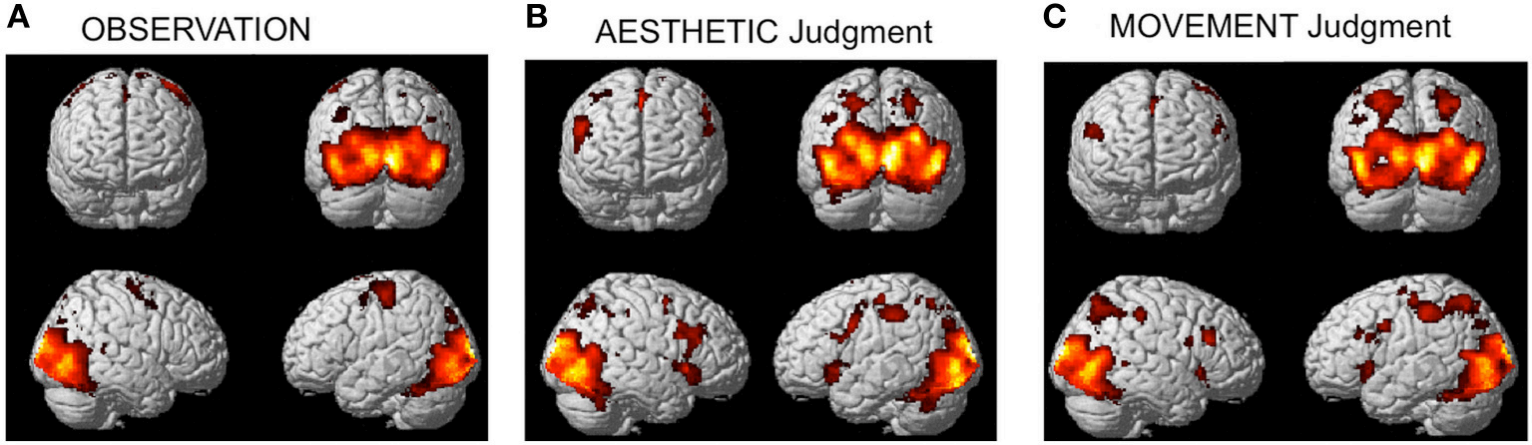
**Activations observed during (A) observation (B) aesthetic judgment, and (C) movement judgment tasks vs. implicit baseline across stimulus categories (nature dynamic, nature static, human dynamic, and human static)**. Group-averaged statistical parametric maps are rendered onto the MNI brain template (P_FWEcorr_ < 0.05).

The activation pattern observed for all stimulus-categories (ND, NS, HD, HS) vs. implicit baseline was very similar independently of the experimental task. In particular, activations were observed in visual occipito-temporal areas, medial temporal areas—to include the fusiform, lingual gyri and hippocampus –, the parietal lobe, supplementary motor area (SMA) and dorsal premotor cortex in all three tasks (Figure 3). Additionally, for aesthetic and movement judgment tasks, activations were found in left somatosensory cortex (SI), bilateral ventral prefrontal cortex as well as an extended bilateral insular activation (see Table 1 for coordinates and statistical details).

**Table 1:** **Global activation pattern observed for all stimulus-categories (Nature Dynamic, Nature Static, Human Dynamic, Human Static) vs. implicit baseline for the 3 experimental tasks: observation (OBS), aesthetic judgment (AJ), movement judgment (MJ)**.

**Task**	**Brain structure**	**Side**	**K_E_**	***Z***	**Local maxima (MNI)**		
					**x**	**y**	**z**
OBS	Occipito/Temporal cortex	R/L	16,501	Inf	−16	−100	8
	Cuneus			Inf	18	−98	10
	Calcarine			Inf	4	−86	0
	Hippocampus	L	102	7.13	−22	−28	−4
	Precentral gyrus	L	104	6.53	−40	−26	66
	Precentral gyrus	R	146	6.28	38	−12	70
	Parietal cortex	L	77	6.34	−36	−84	40
	SMA	L	169	5.84	−2	8	54
AJ	Occipito/Temporal cortex	R/L	18,847	Inf	16	−98	10
	Cuneus			Inf	16	−98	10
	Calcarine			Inf	12	−90	10
	Hippocampus	R	89	6.13	22	−28	−6
	SMA	R/L	870	7.40	8	16	46
				7.18	−2	12	52
	Insula	L	510	7.37	−32	22	−4
				5.17	−40	8	2
	Insula	R	589	7.26	32	24	−4
				5.26	46	8	2
	Superior parietal lobe	R	293	6.61	24	−66	54
	Ventral prefrontal cortex	R	587	5.66	58	18	30
				5.56	52	30	34
				5.53	54	24	18
MJ	Occipito/Temporal cortex	R/L	17,186	Inf	18	−96	12
	Cuneus			Inf	14	−100	6
	SMA	R/L	490	7.05	6	18	46
					−6	6	56
	Insula	L	369	6.15	−30	26	0
					−30	24	−8
	Insula	R	264	6.21	32	24	2
	Precentral gyrus	L	155	5.40	−48	8	36
	Ventral prefrontal cortex	L	45	5.47	−52	26	30
	Ventral prefrontal cortex	R	222	5.88	56	28	28

The results are thresholded at p < 0.05 family wise error (FWE) corrected at the cluster level (cluster size estimated with a voxel-level threshold of p-uncorrected = 0.001).

#### Observation task

**Figure 4:**
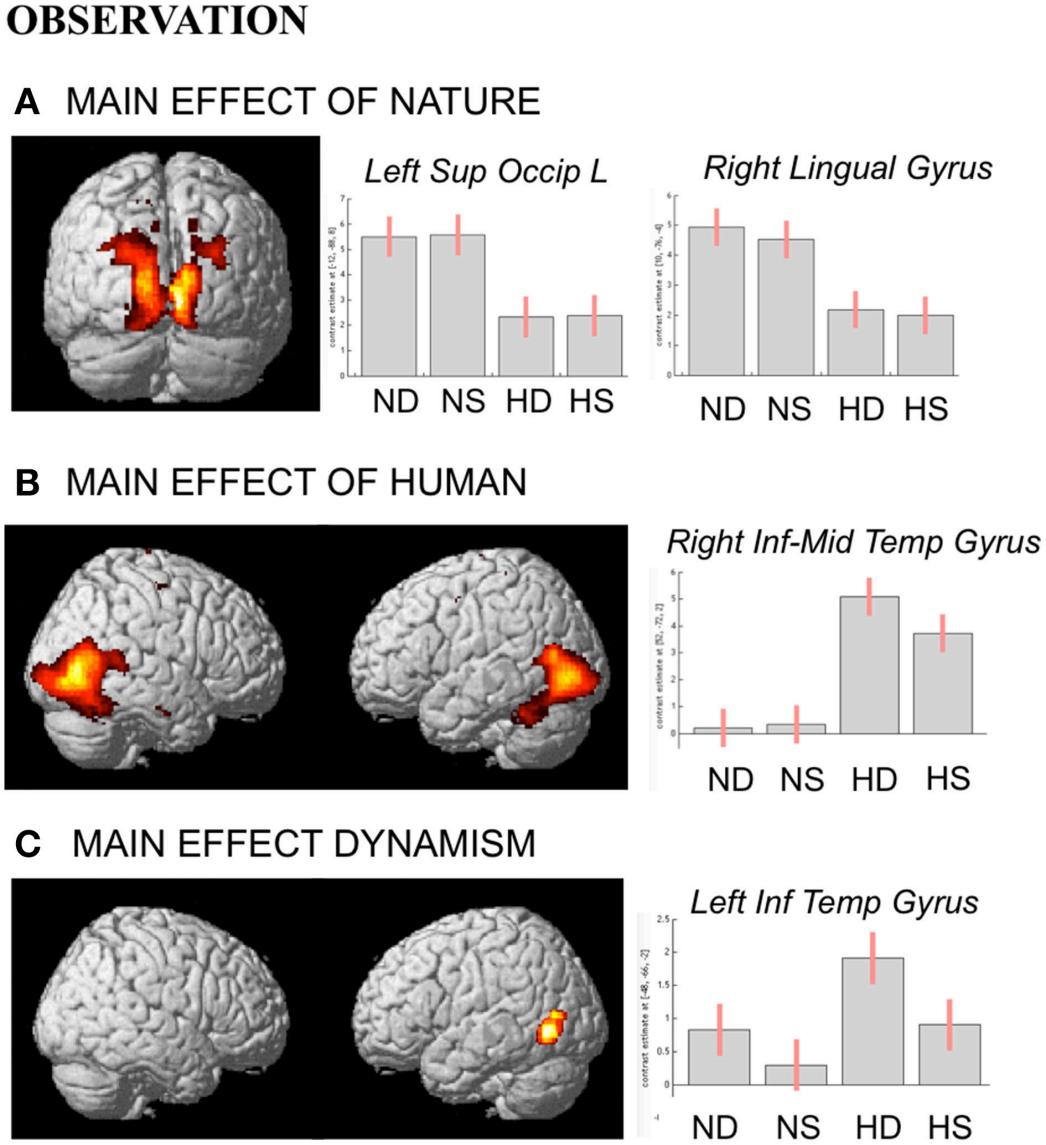
**Activations during observation task for the contrast (A) nature vs. human, (B) human vs. nature, and (C) dynamic vs. static**. The plots show the activity profile for Nature Dynamic (ND), Nature Static (NS), Human Dynamic (HD), and Human Static (HS) in arbitrary units (a.u), +/2 10% confidence intervals (P_FWEcorr_ < 0.05). Group-averaged statistical parametric maps are rendered onto the MNI brain template (P_FWEcorr_ < 0.05).

During observation task, we measured brain activity as a function of stimulus-content (nature, human) and stimulus-dynamism (dynamic, static). The results revealed, for nature vs. human content stimuli, enhanced activation in occipital and posterior parietal areas (to include the cuneus-precuneus), whereas the opposite contrast (human vs. nature) produced activation in inferior and middle temporal sulci to include the lateral occipital complex (LOC) extending to the extrastriate body area (EBA), superior temporal sulcus (STS), and medio-temporal (MT) complex bilaterally and in the precuneus. Dynamic stimuli evoked a stronger activation in left inferior temporal sulcus than the static images (Figure 4). Simple effects contrast analyses revealed that EBA activation, bilaterally, was driven by the dynamic human paintings. See Table 2 for coordinates and statistical details.

**Table 2:** **Contrast analysis as a function of stimulus content (human—H, nature—N) and stimulus dynamism (dynamic—D, static—S) during OBSERVATION task**.

**Contrast**	**Brain structure**	**Side**	**K_E_**	***p.* FWE corr cluster level**	***Z***	**Local maxima (MNI)**		
						**x**	**y**	**z**
N vs. H	Lingual gyrus	R/L	6998	0.000	Inf	10	−76	−4
					7.24	−10	−84	−4
	Calcarine	L/R			7.80	−12	−76	−6
					7.77	14	−86	10
	Sup occipital cortex	L/R			7.48	−12	−88	8
					3.39	16	−86	32
H vs. N	Inf/Mid temp sulcus-EBA	R	3540	0.000	Inf	52	−72	2
	MT complex				Inf.	46	−80	−6
	Fusiform				6.96	42	−48	−24
	Inf/Middle temp sulcus	L	2412	0.000	Inf	−44	−82	−2
	MT complex				Inf	−44	−82	−2
	EBA				6.76	−54	−72	12
D vs. S	Inf. temporal gyrus	L	645	0.000	5.07	−48	−66	−2
HD vs. HS	Inf. temporal gyrus	L	608	0.000	4.88	−48	−66	−2
	Inf. temporal gyrus	R	276	0.012	4.33	54	−58	−8

The results are thresholded at p < 0.05 FWE corrected at the cluster level.

#### Aesthetic judgment task

**Figure 5:**
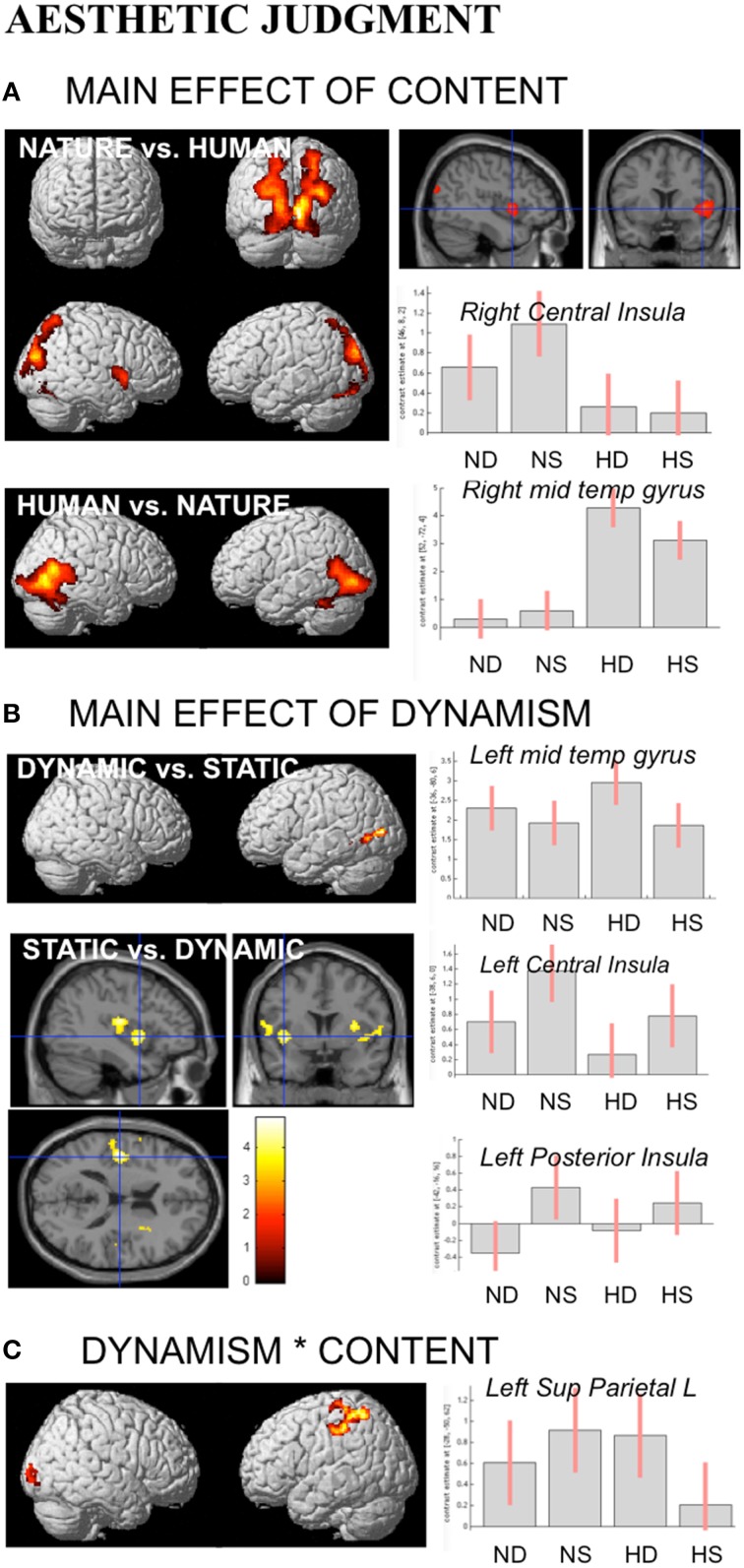
**Activations during Aesthetic Judgment task as a function of (A) stimulus-content (nature, human), (B) stimulus-dynamism (dynamic, static), and (C) the interaction effects between content and dynamism**. The plots show the activity profile for Nature Dynamic (ND), Nature Static (NS), Human Dynamic (HD), and Human Static (HS) stimuli in arbitrary units (a.u), +/2 10% confidence intervals (P_FWEcorr_ < 0.05). Group-averaged statistical parametric maps are rendered onto the MNI brain template (P_FWEcorr_ < 0.05). “^*^” Stems for “interaction.”

During aesthetic judgment task, in line with the activations found for observation task, nature vs. human content stimuli produced enhanced activation in occipital and posterior parietal areas. An additional activation was observed in right central insula. The opposite contrast, human vs. nature, produced activation in inferior and middle temporal sulci to include EBA bilaterally, STS and MT complex, as well as the fusiform gyrus bilaterally (Figure 5A).

With respect to the effect of dynamism on brain activations, dynamic stimuli evoked a stronger activation, compared to the static images and independently of stimulus content, in middle temporal sulcus, whereas static stimuli evoked activation in central and posterior insular cortex bilaterally (Figure 5B). Simple contrast analyses showed that temporal activation was mainly evoked by dynamic compared to static human stimuli, whereas posterior and central insula activations were largely produced by the static compared to dynamic nature stimuli (see plots in Figure 5B).

Additionally, interaction analyses revealed a quite extensive enhanced activation in left parietal lobe, including the somatomotor cortex and superior parietal lobule, and right occipital/calcarine cortex, with dynamic human and static nature stimuli producing greater activation than static human and dynamic nature images, respectively (Figure 5C). Simple effects contrast analyses showed a significant difference in PL only between human dynamic vs. human static stimuli.

See Table 3 for coordinates and statistical details.

**Table 3:** **Contrast analysis as a function of stimulus content (human—H, nature—N) and stimulus dynamism (dynamic—D, static—S) during AESTHETIC judgment task**.

**Contrast**	**Brain structure**	**Side**	**K_E_**	***p.* FWE corr cluster level**	***Z***	**Local maxima (MNI)**		
						**x**	**y**	**z**
N vs. H	Lingual gyrus	R/L	1300	0.000	Inf	12	−86	−8
					Inf	−8	−72	−4
	Mid/Sup occipital cortex	R/L			7.20	30	−82	20
					7.33	−12	−90	6
	Superior parietal lobe	R/L			6.16	24	−66	54
					4.95	−16	−70	50
	Central insula	R	284	0.009	5.01	46	8	2
H vs. N	Inf/Mid temp sulcus—EBA	R	3768	0.000	Inf	52	−72	4
	Fusiform				7.16	42	−44	−24
	Inf/Mid temp sulcus—EBA	L	2214	0.000	Inf	−44	−84	−2
	Fusiform				6.35	−40	−46	−26
	Precuneus	R	416	0.001	4.85	4	−62	36
D vs. S	Middle temporal sulcus	L	272	0.011	4.04	−36	−80	6
S vs. D	Cen/Post insula	L	747	0.000	4.34	−44	−16	16
					4.68	−38	6	0
	Cen/Post insula	R	711	0.000	4.28	56	0	6
					4.22	40	12	−4
Content^*^Dynamism	Postcentral gyrus	L	831	0.000	4.18	−30	−30	70
	Superior parietal lobule	L			4.00	−28	−50	62
	Occipital/Calcarine cortex	R	185	0.000	3.88	14	−100	4
NS vs. ND	Cen/Post insula	L	804	0.000	4.34	−42	−14	16
					4.07	−36	−20	4
HD vs. HS	Posterior temporal cortex	L	729	0.000	4.47	−38	−82	6
	Posterior temporal cortex	R	452	0.000	5.39	52	−58	−6
	Posterior parietal cortex	L	518	0.001	4.49	−30	−54	64

The results are thresholded at p < 0.05 FWE corrected at the cluster level.

“*” Stems for “interaction.”

#### Movement judgment task

**Figure 6:**
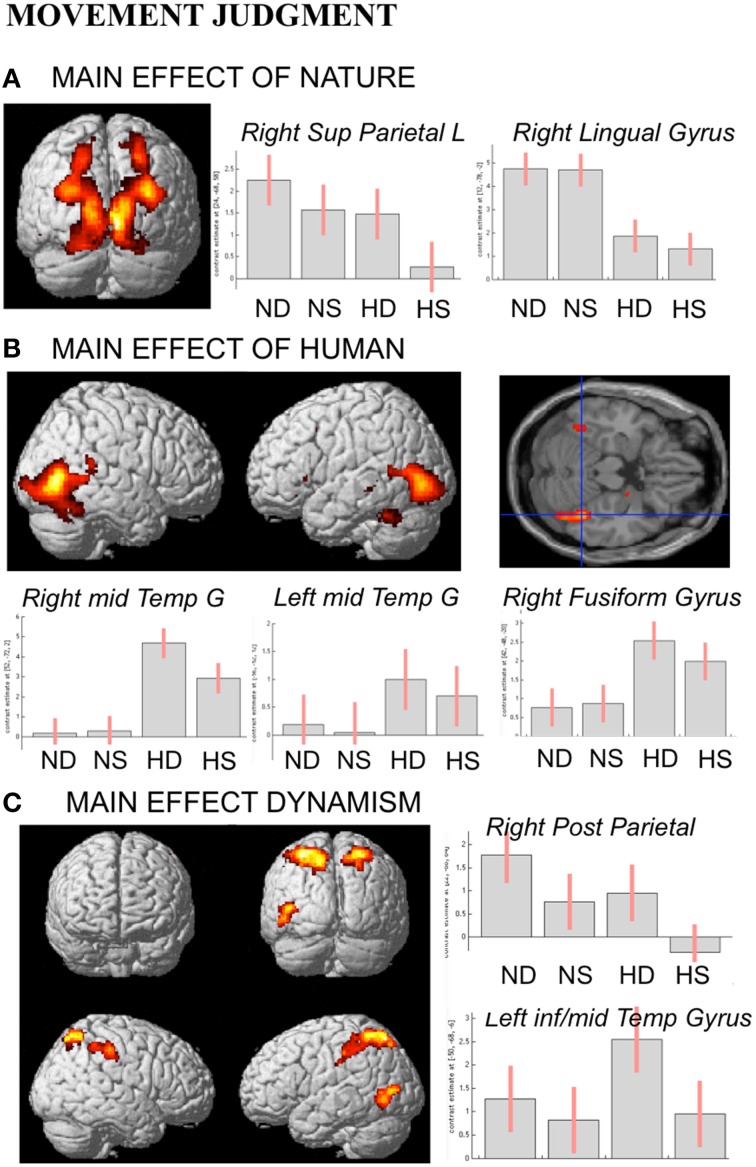
**Activations during Movement Judgment task for the contrast (A) nature vs. human, (B) human vs. nature, and (C) dynamic vs. static**. The plots show the activity profile for Nature Dynamic (ND), Nature Static (NS), Human Dynamic (HD), and Human Static (HS) stimuli in arbitrary units (a.u), +/2 10% confidence intervals (P_FWEcorr_ < 0.05). Group-averaged statistical parametric maps are rendered onto the MNI brain template (P_FWEcorr_ < 0.05).

In line with activations found for OBS and AJ tasks, during movement judgment task enhanced signal change was observed in bilateral occipital and posterior parietal areas for nature vs. human content stimuli, whereas the opposite contrast produced activation in inferior, middle temporal sulci and in fusiform gyrus bilaterally (Figures 6A,B).

With respect to the contrast dynamic vs. static stimuli, greater brain activation was observed for dynamic compared to static images in posterior parietal and intraparietal sulcus bilaterally, as well as in left inferior-middle temporal sulcus (Figure 6C). Simple effects contrast showed that these activations were mainly driven by judgment of the human dynamic compared to the human static paintings. The opposite contrast, i.e., static vs. dynamic paintings, produced no significant activations.

See Table 4 for coordinates and statistical details.

**Table 4:** **Contrast analysis as a function of stimulus content (human—H, nature—N) and stimulus dynamism (dynamic—D, static—S) during MOVEMENT judgment task**.

**Contrast**	**Brain structure**	**Side**	**K_E_**	***p.* FWE corr cluster level**	***Z***	**Local maxima (MNI)**		
						**x**	**y**	**z**
N vs. H	Lingual gyrus	R/L	12,090	0.000	Inf	12	−78	−2
					Inf	−8	−70	−4
	Calcarine cortex	R			Inf	12	−86	4
	Superior parietal lobule	L			5.32	−24	−62	42
		R			4.43	24	−68	58
H vs. N	Inf/Mid temp sulcus—EBA	R	4207	0.000	Inf	52	−72	2
	Fusiform				6.55	42	−48	−20
	Inf/Mid temp sulcus—EBA	L	2191	0.000	Inf	−46	−82	−4
D vs. S	Posterior parietal cortex	R	690	0.000	5.50	22	−68	64
	Intraparietal sulcus	R	536	0.000	4.37	50	−28	48
	Posterior parietal cortex/Intraparietal sulcus	L	1872	0.000	4.93	−30	−56	62
	Inf/Middle temporal sulcus	L	536	0.000	4.37	−50	−68	−6
Content^*^Dynamism	Middle occipital cortex	R	421	0.000	3.11	26	−88	4
HD vs. HS	Posterior parietal cortex/Intraparietal sulcus	L	1450	0.000	4.76	−30	−60	60
	Posterior parietal cortex/Intraparietal sulcus	R	837	0.000	4.95	30	−56	62
	Inf/Mid temp sulcus—EBA	L	1081	0.000	5.11	−40	−74	6
		R	760	0.000	4.61	28	−88	6
	Inferior frontal gyrus	L	472	0.001	4.43	−38	42	4

The results are thresholded at p < 0.05 FWE corrected at the cluster level.

“*” Stems for “interaction.”

## Discussion

The neural underpinnings of movement perception and its contribution to the aesthetic experience have been often described in association with representations of human subjects; however, no such specificity has been defined in previous neuroimaging studies with respect to contents that lack a human form. The aim of the present work was to clarify the effects of perceived movement as a function of stimulus content on the aesthetic processing of artworks. For this purpose, participants, without formal training in arts, viewed representational paintings depicting human figures compared to nature scenes categorized as dynamic and static. The stimuli were presented in three tasks: observation, aesthetic judgment, and movement judgment. Observation and movement judgment tasks were introduced, alongside aesthetic judgment, to better outline the nature of the activations observed in AJ.

A global analysis of the imaging data revealed, independently of task and stimulus type, activations (vs. baseline) of visual occipito-temporal areas, medial temporal areas to include the fusiform, lingual gyri, and hippocampus. These structures are involved in the perceptual analysis, implicit memory integration and explicit classification of the stimulus (Leder et al., 2004; see also Leder and Nadal, 2014; Leder et al., 2015). Furthermore, activations were observed in the parietal lobe, SMA and dorsal premotor cortex and, for aesthetic and movement judgment tasks, in left primary somatosensory cortex, ventral prefrontal cortex bilaterally as well as in the anterior insula bilaterally. Activations found for the two judgment tasks in motor-related structures independently of stimulus categorization suggest that, in general, aesthetic judgment is related to movement perception. At a low level of processing, the relationship between movement and aesthetics was shown in a recent TMS study (Cattaneo et al., 2015), in which triple-pulse TMS was applied over the visual motion-sensitive area V5. TMS stimulation determined a decrease in the participants' perceived sense of motion of abstract and representational paintings (see also Thakral et al., 2012). Interestingly, decreased liking after TMS stimulation was observed only for the abstract, and not the representational, paintings. This finding supports the idea that sensory regions are involved in the aesthetic process when attention is focused on low-level features, as in the case of, at least some, abstract works. For representational paintings, in which content is given, the link between movement and aesthetics may, on the other hand, involve high-order processing, as suggested by the activation of the cortical motor-related structures described here.

### Human content paintings

Contrast analyses within each task (OBS, AJ, MJ) showed that, for all tasks, the presentation of paintings portraying a human subject produced a stronger activation, compared to nature content stimuli, of the precuneus (midline section), inferior and middle temporal sulci, including EBA, STS, MT complex and of the fusiform gyrus bilaterally. This latter activation was expected. It is known from both monkey (see Desimone et al., 1984; Tsao et al., 2006; Gross, 2008) and human studies that portions of the inferotemporal lobe and of its human homolog (the fusiform gyrus), play a crucial role in the processing of faces (for review, see McKone and Kanwisher, 2005; Gross, 2008). Furthermore, it was shown that some sectors of the fusiform gyrus encode, with nearly the same level of selectivity, images of human bodies (Peelen and Downing, 2005; Schwarzlose et al., 2005). Therefore, fusiform activation observed in the present study likely reflected a detailed visual analysis of the physical aspects of the body. Its activation observed also during MJ task further suggests that the fusiform gyrus may be involved in the processing of body configurations portraying actions and implying movement. Action processing may, at this level, also convey information about the agents' emotional state (e.g., anger; Hadjikhani and de Gelder, 2003), contributing to the building up of the affective component of aesthetic processing for artworks representing human contents. Although not explicitly described, this affective component could be integrated within the hippocampus-centered affect system proposed in Koelsch et al.'s (2015) model. Fusiform activation found also during observation task further suggests that these mechanisms are evoked quite spontaneously in the viewer.

The part of the precuneus found activated for human relative to nature content stimuli is its midline section, as opposed to the more dorsal part observed for nature content stimuli. As part of the cortical midline structures, precuneus activity has been suggested to be associated with episodic memory retrieval and even with what has been referred to as the “self” (for review see Cavanna and Trimble, 2006). Episodic memory is employed for storage and recall of previously experienced events and has autobiographical reference (Tulving, 1983, 2002) since it involves the recollection of information that is linked to an individual's personal experiences. Additionally, memory-related imagery has been often associated with bilateral activation of the anterior precuneus, reinforcing the hypothesis that the precuneus plays a key role in visual imagery that occurs during episodic memory recall (Buckner et al., 1995; Fletcher et al., 1996; Halsband et al., 1998). In Di Dio et al. (2007), the precuneus was found activated during the presentation of Classical sculpture images representing the canonical human body structure as opposed to modified versions of the same body structures. This finding suggested that precuneus activation, alongside prefrontal activations, could be the result of a match between an inner representation of a standard body with the observed canonical images. In the present study, enhanced precuneus activation for human content paintings was possibly triggered by association between the portrayed subject and self-referenced episodes or even between the portrayed action and specific motor configurations, as supported by its activation also during the MJ task.

### Effect of dynamism on temporal and parietal activations for human content paintings

Contrast analyses carried out for human content paintings showed that posterior temporal activations were mainly evoked by paintings portraying a dynamic human subject. Data from neuroimaging studies indicate that the human body is visually described in the extrastriate body area (EBA, e.g., Calvo-Merino et al., 2010), which borders and, in some instances, overlaps with the lateral occipital complex (LOC; Malach et al., 1995; Grill-Spector et al., 2001). The LOC and the temporal visual areas are known to respond to the presentation of body parts or even to the whole human body (Downing et al., 2001; Astafiev et al., 2004). Additionally, enhanced activation of STS for paintings depicting a dynamic human subject was likely due to the representation of body movement encoded in this region (see Perrett et al., 1989; Allison et al., 2000; Pelphrey et al., 2004; Thompson et al., 2005). STS, in fact, is involved in processing movement of different body parts and is shown to respond also to the presentation of static stimuli that imply motion. A similar functional property was also reported for area MT/V5 (Kourtzi and Kanwisher, 2000; see also Proverbio et al., 2009; Thakral et al., 2012). Comparing cerebral activity between paintings showing dynamic and static human subjects showed, in fact, enhanced activation of MT/MST complex bilaterally for the dynamic images (e.g., Kolster et al., 2010).

Noteworthy was also the activation, in the same contrast (dynamic vs. static human paintings), of bilateral posterior parietal lobe—including SPL and IPL—during AJ and MJ tasks, though broader for MJ task. SPL is a crucial area for sensorimotor integration. Its lesions induce both sensory and motor deficits consistent with an inability to maintain an updated internal representation of the body state (Wolpert et al., 1998). Functionally, SPL is involved in action imitation (e.g., Grezes, 1998; Krüger et al., 2014) providing a kinaesthetic blueprint during movement observation (Krüger et al., 2014). Likewise, IPL activation is often associated with action understanding and imitation (Buccino et al., 2004; see also, Buccino et al., 2001; Molenberghs et al., 2012; Rizzolatti and Fogassi, 2014). It is likely that these parietal activations in our study were affected by the intrinsic dynamic properties of the represented human subject—categorized as dynamic—and the sense of movement evoked in the observer (see Freedberg and Gallese, 2007). It is worth noting that paintings were displayed in their entirety and the human actions were surrounded by a pictorial context. Therefore, increased activation of the parietal cortex for dynamic human paintings during both aesthetic and movement judgment tasks suggests that participants paid particular attention to the depicted action. This idea is supported by our previous eye-tracking studies (Massaro et al., 2012; Savazzi et al., 2014) showing that, when appraising the dynamic human content paintings, the participants focused their attention on the limbs portraying the subject's action.

### Nature content paintings

The comparison between aesthetic evaluations of nature as opposed to human content paintings revealed that viewers generally preferred nature scenes. This finding was supported by the aesthetic evaluation of paintings from our former psychophysical work (Savazzi et al., 2014). Preference for nature scenes may follow various tentative explanations since there is not a universally accepted theory for the aesthetics of natural environments (Maulan et al., 2006). In our former work, we hypothesized that this preference was possibly due to the ever-contemporary depiction of nature that, compared to the human figure, remains less influenced by the changing of times. However, other interpretations remain open. The biophilia hypothesis (Wilson, 1984), for example, suggests that preference for nature is grounded in humans' inherent need for affiliation with natural environments and other forms of life. Hereditary inclination toward establishing an emotional bond with nature and other livings may stem from the fact that, during evolution, certain rewards or advantages associated with natural settings were crucial for survival (Ulrich, 1993). Humans' positive responses to natural settings might be then influenced by biologically prepared learning (Ulrich, 1993). Additionally, Bourassa (1988) describes two principles for landscape aesthetics: biological and cultural. The biological principle asserts that aesthetic pleasure in landscapes derives from the dialectic of refuge and prospect, a theory that proposes that human beings experience pleasure and satisfaction with landscapes that respond to their biological needs. These and other interpretations for the general preference observed for nature scenes would need further exploration. Within the present data, a *post-hoc* assessment of the stimuli on a few dimensions that have been previously shown to correlate with aesthetic preference, including stimulus valence and arousal, revealed that nature stimuli received higher scores than human content paintings. This is in line with Jacobs and colleagues' results showing that words describing phenomena from nature (animals, flowers, rainbow, etc.) were rated high on beauty, valence, and imageability (Jacobs et al., 2015). These factors, alongside others related to unexplored perceptual dimensions of stimulus processing such as the sensori-motor dimension described as the desire to approach, avoid, explore, touch, etc., may provide perceptive explanations for the aesthetic preference often observed for nature content stimuli. In the present study, we did not explicitly control for these or other factors and, therefore, cannot offer grounded explanations for the aesthetic preference ascribed to nature scenes compared to human content paintings. Our imaging data, however, provide results with respect to the neural underpinnings associated with the aesthetic processing of nature scenes upon which insightful considerations can be drawn.

Contrast analyses between nature and human content stimuli during the aesthetic judgment task showed enhanced activation, for nature scenes, in occipital cortex, medial temporal areas, including the lingual gyrus, the posterior parietal cortex (PPC), including the cuneus/precuneus, and right central insula. Enhanced activation of the visual area was likely due to a thorough visual analysis in which the viewer was engaged during the presentation of nature scenes (shape, color, visual complexity, etc.; e.g., Arnheim, 1992; Zellner et al., 2010), as also pinpointed in our eye-tracking works (Massaro et al., 2012; Savazzi et al., 2014). Moreover, activation of the lingual gyrus, a structure involved in the visual analysis of complex images, has been previously reported in association with exploration of paintings and portraits of nature scenes (Kawabata and Zeki, 2004; Vartanian and Goel, 2004; Mizokami et al., 2014; for a review, see Vartanian and Skov, 2014). In the exploration of pictorial stimuli, the PPC/precuneus have been often associated with visuospatial exploration (see Fairhall and Ishai, 2008; Cupchik et al., 2009) and, in Silveira et al. (2012), their activation was found from the comparison between naturalistic and surrealistic content paintings. In line with the idea that these structures are also involved in the coding of episodic memory (Sestieri et al., 2010), PPC/precuneus activation for nature scenes could possibly be due to a matching process between specific aspects of real environments and the observer's memory of them. Additionally, the posterior parietal cortex holds a pragmatic representation of movement, given that this area is a site of convergence between visual and motor input. This functional role, which is supported by its activation also during MJ task, further suggests that the aesthetic evaluation of nature scenes involves a motor component.

### Effect of dynamism and insular activation for nature content stimuli

While the constellation of activations found in the present study for both nature and human content paintings reflects activation patterns commonly described for these categories of stimuli, one activation deserves special consideration as a novel result of this study: namely, activation of central and posterior insula during AJ task. More specifically, during the aesthetic evaluation of nature stimuli, we found right central insula activation for nature compared to human content paintings and enhanced activation of central and posterior thirds for static nature compared to dynamic nature paintings. Contrarily to most activations described here, central and posterior insular activations were found only during AJ task, suggesting its specific involvement in the explicit evaluation of the stimuli aesthetics. What could be the role of the second and third sectors of the insula in the aesthetic processing of nature scenes, and in particular of the static ones?

Single neuron studies show that central insula is endowed with sensorimotor properties (Robinson and Burton, 1980; Schneider et al., 1993; see also Jezzini et al., 2012) given that it is connected with the somatosensory cortex (e.g., Mishkin, 1979; Friedman et al., 1986; Augustine, 1996; Caruana et al., 2011). Additionally, physiological and anatomical data show that the monkey's dorso-central insula is connected with area AIP (Borra et al., 2008), area F5 (Gerbella et al., 2010), and area 12r (Borra et al., 2011) suggesting that this sector is part of the motor circuit related to the organization of, at least, arm movements (Jeannerod et al., 1995; Nelissen and Vanduffel, 2011; Jezzini et al., 2012; Rizzolatti et al., 2014). In agreement with these findings, there are data showing that the electrical stimulation of the middle and posterior short gyri of the insula determined evoked potential in the precentral gyrus and in the superior and inferior parietal lobule (Almashaikhi et al., 2014a) demonstrating strict connections between areas with motor properties and the insula. Furthermore, recent findings (Di Cesare et al., 2013, 2015) show that the central insula specifically codes the style of movements, i.e., how the action is performed (vitality affects; Stern, 1985, 2010). The style of movement would be influenced, according to the theory, by the affective state of the individual through connections with medial temporal areas (Di Cesare et al., 2013; see also Löken et al., 2009; Morrison et al., 2011). Furthermore, Di Cesare et al. (2015) showed that central insula responds both during observation and execution of different action styles. This new functional description of the central insula suggests that its activation is important in the modulation of movement kinematics enabling an individual to act, for example, gently or rudely in accordance with his/her emotional state and to understand the emotion states of others through observation of movement kinematics. In this light, within the present study, central insula may represent an important anatomical locus where the internal affective state of the individual interacts with sensori-motor processes during the aesthetic evaluation of nature content paintings.

The central insula was shown to strongly connect with the posterior insula (Almashaikhi et al., 2014b). The posterior third of the insular cortex (pIC), also labeled as primary interoceptive cortex, is described as a crucial region for interoception (Craig, 2003) receiving a direct representation of homeostatic afferent information from thalamocortical pathways and engendering distinct bodily or interoceptive feelings by projections onto the anterior insula for an emotional evaluation (Craig, 2003; see also Saper, 2002). With respect to the cutaneous senses, pIC could constitute the primary cortical locus of an interoceptive system regulating affective feeling states from the skin such as pain, warmth, itch, and sensual touch (e.g., painful stimulation; Craig, 2003; pleasant stroking; Björnsdotter et al., 2009; Löken et al., 2009). Additionally, it has been repeatedly demonstrated that the mid-posterior insula processes different somato and viscerosensory stimuli (Peyron et al., 2000, 2002; Ostrowsky et al., 2002; Dupont et al., 2003; Shelley and Trimble, 2004; Chen, 2007; Kurth et al., 2010a,b). It was suggested that pIC, constituting a central cortical node in a system forming a neural representation of the “material me” (Craig, 2003), that in psychoanalytical terms could be indented as the me-skin (moi-peau; Anzieu, 1985), could be part of an inhibitory mechanism enabling the observer to distinguish, at the phenomenal level, to whom the observed feelings belong, activating when being touched and deactivating when observing somebody else being touched (Ebisch et al., 2011; see also Ebisch et al., 2013). Interestingly, movement was elicited by electrical stimulation of this region in humans (Showers and Lauer, 1961) and its stimulation was shown to raise a sensation or urge of movement (Penfield and Faulk, 1955).

These physiological, anatomical and functional descriptions of the posterior and central insula offer a tentative interpretation for their role in the aesthetic processing of artworks representing static nature scenes and, more generally, static pictorial representations, as suggested by the main effect of dynamism showing that central and posterior insulae were activated bilaterally for static relative to dynamic paintings, independently of stimulus content. During the explicit aesthetic evaluation of a painting, insula activation could subserve processes favoring a first person sensorimotor experience *necessary* to judge whether a static image is more or less beautiful, independently of the end-result of this aesthetic analysis. According to this view, the observer would be engaged by the represented scene, immersed in the portrayed context in an “embodied” manner. With respect to nature scenes, embodiment would imply participation in the first person of the observing individual, for whom actions and sensations are recalled by his/her proclivity to explore the represented territory, not only visually or through mental imagery, but also with his/her body through movement and touch. The visuo-spatial “navigation” and motor coding supported by the parietal activation in Cela-Conde et al. (2009) and highlighted by the interaction effect observed in the present study during the AJ task would then find an affective sensori-motor complement in insular activation. The interaction effect found in the somatomotor region and superior parietal lobule, in fact, contrary to our predictions, highlighted enhanced activation for static compared to dynamic nature paintings, whereas the opposite pattern of activation was observed for human content paintings, with dynamic stimuli activating PL more that the static ones. How can these apparently contradictory results be explained?

Embodied/motor processes are largely recognized for human content paintings trough activation of mirror and mirror-like areas (premotor, parietal, and superior temporal areas), as also found in the present study. When a human being is represented in a painting, the visible aspects of behavior (actions and emotions) would resonate with the viewer's motor experiences framing the beholders' involvement with the represented actions, which are best described in dynamic human portrayals. For nature content paintings, since there is no action description, actions are boundless and the freedom to explore in the first person gathers another, more ample, imaginative valence. In line with this idea, Kaplan and Kaplan's (1978, 1989) information processing theory on landscape preferences states that aesthetics reflects the functional potential of spaces, since we value environments with promising information for exploration, comprehension, and feeling safe (i.e., people want to explore safely by seeking information and at the same time look for new challenges). This idea is also in line with Berlyne's (1971) view that environmental perception is a process of exploratory behavior and information transmission, as well as with Kaplan and Kaplan (1989), who outlined some predictors of preference. Among these, are complexity (that keeps one's attention and desire for exploration); readability (i.e., easiness of exploration of a territory); and mystery (that drives toward exploration and interaction with the environment). These views are congruent with greater activation observed both in somatomotor areas and the insula, which are strictly connected as described above, when attending to static nature stimuli that, in this study, mostly depicted landscapes, and namely environments that would favor exploratory behavior, against dynamic nature scenes mostly portraying falls and seas.

### Conclusions

In general, our results show that the aesthetic judgment of human and nature content paintings involves a motor component processed, in both instances, by our cortical motor system through activation of parietal and premotor areas. While human content paintings, particularly the dynamic ones, determine a motor resonance most likely evoked by the depicted actions, the aesthetic processing of nature content paintings representing landscape scenarios would involve an additional sensori-motor component internally generated to favor imaginary exploratory behavior. In this stance, aesthetic processing requires a sort of immersion process within the represented scene on the basis of the beholder's own experiences, needs and emotions.

The motor component of aesthetic perception has been poorly described or even neglected by the current theories of aesthetic processing. This work provides new evidence suggesting, not only that movement perception plays a significant role in aesthetic processing, but also that the way in which this component is involved in the explicit aesthetic evaluation of an artwork depends on the stimulus content. In this respect, it is important to note that the distinction made a priori between static and dynamic nature stimuli uncovered a new factor that appears to affect aesthetic processing when analyzing the content of a painting, i.e., “motor” accessibility. For its relevance, studying the independent and interactive effects of movement perception and content accessibility on aesthetic processing would require furher investigation.

## Future developments: A link between visual aesthetics and literature reading

The interpretation of the present results may find a parallel with the very recent theoretical approach to the empirical studies involved in literature reception and immersive reading, thus inviting to a more comprehensive and integrated approach to the study of aesthetics. Although brain imaging results on literature reading do not reflect the activated regions in the present work (Hsu et al., 2014), within this domain it was suggested that immersive experiences are facilitated by the motor component of affective empathy, at least for materials, which feature particularly vivid descriptions of the behavioral (expressive) aspects of emotion (Wojciehowski and Gallese, 2011; Jacobs, 2015b). In this respect, two distinct processes can be outlined, which encompass the notions of background and foreground features (Jacobs, 2015b). Background features (e.g., familiarity and situational embedding) would involve motor processes associated with the immersion of the reader in content (Wojciehowski and Gallese, 2011; Jacobs, 2015a). In this respect, Lehne et al. (2015) found that the rated amount of actions reported in story segments highly correlates with immersion ratings, suggesting that fiction feelings supported by scenes full of actions facilitate immersive processes (see also, Hsu et al., 2014; Lüdtke et al., 2014). Relating these findings to the field of visual aesthetics, it can be reasonably posited that, starting from posterior parietal/precuneus activation, specific processes are set (memory-, spatial-, and motor-related), which would allow the receiver (reader or art viewer) to be engaged in an immersive or, otherwise termed, transportation experience characterized by a full involvement of the recipient in a story (or image)—see also Burke (2015). These processes are hypothesized as part of an implicit system of embodied simulation, which is potentiated by the fact that, most of the time, the recipient is motionless when beholding an artwork or reading (“liberated embodied simulation”; see Wojciehowski and Gallese, 2011). Foreground features, on the other hand, are hypothesized as part of an explicit system underlying the aesthetic feeling associated with a specific content. In line with this idea, foreground and background feature processing may altogether shape the theorized notion of “disportation,” which encompasses the concepts of felt movement (not actual movement) and positive emotion (Burke, 2015). These ideas, conceptualized for literature reading, bring to attention the crucial role of the motor and affective components in immersive, embodied processes, here largely described for visual aesthetics. Using lyric poetry, which has a strong emotional component and makes use of imagery, especially of nature, Lüdtke et al. (2014) further support this theoretical approach, according to which stimuli describing natural scenes can elicit an emotional state syntonic with the represented environment through the involvement of “embodied” motor mechanisms (see also Burke, 2011).

## Limitations and outlook

One of the main issues that the present study and experimental aesthetics in general has to deal with is the problem of stimulus selection. When employing paintings that differ in many dimensions (color, form, content, spatial relations between parts, style, just to cite a few) complete control is very challenging. Moreover, whereas much control may diminish the ecological and generalization power of the results, the use of real artworks may reduce the power of hypothesis testing. In the present study, we reported ratings on some of the dimensions that are shown to correlate with aesthetic processing (familiarity, complexity, valence, arousal, etc.). Some of these factors could, at least partially, explain the aesthetic preference observed for nature paintings. However, since there was no full control over the possible sources of variation contributing to aesthetic preference, our main findings and interpretations were focused on the brain activations observed during AJ irrespective of aesthetic preference.

Another point we would like to raise is that particular care should be given to the interpretation of results drawn from empirical aesthetics when dealing with aesthetic judgments vs. aesthetic experience. As previously observed (e.g., Cupchik and Laszlo, 1992; Leder et al., 2004; Di Dio et al., 2007; Höfel and Jacobsen, 2007) aesthetic experiences arise as the result of aesthetic appreciation of an artwork when the contextual conditions are set, whereas aesthetic judgments force the individuals to recruit cognitive resources that may overshadow the activation of areas involved in the aesthetic experience, such as emotion processing areas. It is not easy to promote an aesthetic experience in an experimental environment. In this attempt, at least two strategies could be employed: (1) define a proper mental set, i.e., setting the conditions to promote the emergence of the aesthetic experience (e.g., task); (2) the stimuli need to be powerful enough to engage the observers in such an experience even when merely viewing images lying down on a MRI bed. In fact, experience in real art environments may be substantially different (see Brieber et al., 2014). In the present study, we used an observation task to outline processes observed in deliberate aesthetic judgments (AJ) that can be also evoked in an automatic fashion (OBS). With respect to our main finding concerning insula activation for nature content stimuli in the AJ task, we did not find a corresponding activation in OBS. This, though, does not necessarily rule out the idea that the sensori-motor component involved in the aesthetic judgment of nature content paintings can be also spontaneously triggered. Perhaps, a proper contextual frame, such as an art gallery, where the receiver can benefit from the direct contact with the artworks, could be possibly able to evoke such a response.

Finally, this work reports novel findings in the field of experimental aesthetics, which aim at encouraging research toward a thoughtful and focused exploration of variables such as content and movement in the aesthetic processing of artworks. However, as for most exploratory investigations, our interpretations require further support. This is most important if also accounting for the notion of reverse inference (RI). Reverse inference, common, and often neglected in neuroimaging studies, is the phenomenon according to which a cognitive process is inferred from specific brain activations. It is very difficult avoiding this process particularly in experimental aesthetics, a field that is still in its infancy. In consideration of the RI issue, in the present study we cared to specify the stimulus features that may have elicited a particular activation putting forward cautious interpretations based on a broad review of the literature. Best is the use of meta-analyses that, as thoroughly described in Hutzler (2014), may increase the predictive power of RI that, contrarily to the common belief, when properly used can allow research to progress in specific domains.

### Conflict of interest statement

The authors declare that the research was conducted in the absence of any commercial or financial relationships that could be construed as a potential conflict of interest.
