# Supplementary material for: Human, Nature, Dynamism: The Effects of Content and Movement Perception on Brain Activations during the Aesthetic Judgment of Representational Paintings
Source: Front Hum Neurosci. 2016 Jan 12;9:705. doi: 10.3389/fnhum.2015.00705 (PMC4709505; doi:10.3389/fnhum.2015.00705)
Supplement: Supplementary file 1 [file Table1.DOCX]

**Supplementary Table 1S.** **A)** Description of the stimuli: for each stimulus, the category, title, artist, year, collection and content description are reported; **B)** Statistics: for each stimulus, the mean and standard error of the mean are reported for the participants' Aesthetic Judgments (AJ) and Movement Judgments (MJ) collected during the scanning sessions (N=19). Independent raters' mean scores and standard errors (N=20) are reported for complexity, arousal, content valance and emotional valance of each stimulus. ND = Nature Dynamic; NS= Nature Static; HD = Human Dynamic; HS = Human Static

**A) Description of the stimuli**

| **Category** | **Title** | **Artist** | **Year** | **Collection** | **Content description** |
| --- | --- | --- | --- | --- | --- |
| HD | The Flea | Crespi, Giuseppe Maria | 1707-1709 | Galleria degli Uffizi, Florence | Woman seated in the act of washing herself |
|  | Richard Humphreys, the Boxer | Hoppner, John | XVIII sec. | The Metropolitan Museum of Art, NY | Man posing as a boxer |
|  | The Morning Toilet | Steen, Jan | 1665 | Rijksmuseum, Amsterdam | Woman seated in the act of putting on a sock |
|  | At the Mirror | Kersting, Georg Friedrich | 1827 | Kunsthalle, Kiel | Woman in the act of hairstyling mirrored in a toeletta |
|  | The Winnower | Millet, Jean- Franois | 1848 | Musée du Louvre, Paris | Winnower working |
|  | Apollo | Dossi, Dosso | 1524 | Galleria Borghese, Rome | Apollo in the act of playing the violin |
| HS | Portrait of an Artist in His Studio | Géricault, Théodore | 1820 | Musée du Louvre, Paris | Seated man staring |
|  | Marchesa Florenzi | Hess, Heinrich Maria Von | 1828 | Neue Pinakothek, Munich | Seated woman posing |
|  | Old Woman Dozing | Maes, Nicolaes | 1656 | Musées Royaux des Beaux-Arts, Brussels | Seated old woman sleeping |
|  | Ferdinand IV, King of Naples | Mengs, Anton Raphael | 1760 | Museo del Prado, Madrid | Royal boy sitting for a portrait |
|  | Old Woman Seated | Puga, Antonio | XVII sec. | Museo del Prado, Madrid | Old woman seated |
|  | Magdalene | Caravaggio | 1596-1597 | Galleria Doria-Pamphili, Rome | Seated woman in the act of sleeping |
| ND | Le torrent (Tivoli) | Michallon, Achille-Etna | 1818-1821 | Musée du Louvre, Paris | Torrent with small falls |
|  | Scogliera a Sestri Levante | Gignous, Eugenio | 1890 | Private Collection | Rocky cliff on a flat sea |
|  | Drifting Clouds | Friedrich, Caspar David | 1820 | Kunsthalle, Hamburg | Clouds on a green valley |
|  | Fog in the Elbe Valley | Friedrich, Caspar David | 1821 | Nationalgalerie, Berlin | Fog in the Elbe Valley |
|  | The North Sea in Moonlight | Friedrich, Caspar David | 1823-1824 | National Gallery, Prague | Reeves in the sea at night |
|  | The Waterfalls at Terni | Hackert, Jacob Philipp | 1779 | Private Collection | Waterfalls |
| NS | Aurore, novembre | Ballot, Clémentine | 1915 | Centre Pompidou, Paris | Valley in the daylight |
|  | Fontainebleau: Oak Trees at Bas-BrŽau | Corot, Camille | 1832 | The Metropolitan Museum of Art, New York | Trees on a hill |
|  | Bohemian Landscape with Mount Milleschauer | Friedrich, Caspar David | 1808 | Gemäldegalerie, Dresden | Valley on a limpid sky background |
|  | The Watzmann | Friedrich, Caspar David | 1824-1825 | Nationalgalerie, Berlin | Rock with mountains in the background |
|  | The Oaktree in the Snow | Friedrich, Caspar David | 1829 | Nationalgalerie, Berlin | Leavesless tree in the snow |
|  | Rocky Crags at l'Estaque | Renoir, Pierre-Auguste | 1882 | Private Collection | Barren rocky view |

**B) Statistics (mean, SE)**

| **Category** | **Title** | **AJ** | **MJ** | **Complexity** | **Arousal** | **Content valance** | **Emotional valance** |
| --- | --- | --- | --- | --- | --- | --- | --- |
| HD | The Flea | 2.15, 0.24 | 2.35, 0.18 | 3.95, 0.37 | 2.65, 0.25 | 2.5, 0.26 | 2.1, 0.27 |
|  | Richard Humphreys, the Boxer | 1.81, 0.20 | 2.31, 0.18 | 1.55, 0.23 | 1.9, 0.38 | 1.4, 0.34 | 1.6, 0.33 |
|  | The Morning Toilet | 1.91, 0.18 | 2.06, 0.11 | 2.05, 0.24 | 1.6, 0.34 | 2.1, 0.26 | 1.7, 0.24 |
|  | At the Mirror | 2.95, 0.20 | 2.38, 0.17 | 2.65, 0.35 | 2.55, 0.32 | 2.9, 0.31 | 3.45, 0.32 |
|  | The Winnower | 2.22, 0.17 | 2.82, 0.17 | 2.7, 0.34 | 1.95, 0.43 | 2.5, 0.38 | 2.25, 0.38 |
|  | Apollo | 2.71, 0.21 | 2.67, 0.18 | 3.25, 0.31 | 2.5, 0.32 | 2.1, 0.26 | 1.85, 0.22 |
| HS | Portrait of an Artist in His Studio | 2.19, 0.25 | 1.54, 0.16 | 2.3, 0.34 | 1.75, 0.32 | 1.95, 0.25 | 1.45, 0.24 |
|  | Marchesa Florenzi | 2.43, 0.20 | 1.71, 0.21 | 3.75, 0.31 | 2.55, 0.34 | 3.3, 0.35 | 3.45, 0.29 |
|  | Old Woman Dozing | 2.47, 0.24 | 1.46, 0.16 | 2.8, 0.35 | 2, 0.35 | 1.9, 0.31 | 1.8, 0.32 |
|  | Ferdinand IV, King of Naples | 1.97, 0.24 | 1.47, 0.20 | 2.25, 0.32 | 1.25, 0.28 | 2.6, 0.31 | 1.45, 0.28 |
|  | Old Woman Seated | 2.29, 0.25 | 1.53, 0.21 | 2.55, 0.35 | 2.05, 0.28 | 1.05, 0.22 | 0.95, 0.21 |
|  | Magdalene | 1.94, 0.18 | 1.22, 0.09 | 2.55, 0.34 | 2.3, 0.4 | 1.55, 0.28 | 1.85, 0.37 |
| ND | Le torrent (Tivoli) | 3.24, 0.17 | 3.38, 0.20 | 2.25, 0.38 | 4.1, 0.35 | 4.25, 0.27 | 4.6, 0.30 |
|  | Scogliera a Sestri Levante | 3.03, 0.18 | 2.94, 0.18 | 2.2, 0.35 | 3.65, 0.34 | 3.75, 0.31 | 4.1, 0.29 |
|  | Drifting Clouds | 2.82, 0.22 | 2.38, 0.20 | 1.9, 0.27 | 3.05, 0.38 | 3.1, 0.39 | 3.45, 0.42 |
|  | Fog in the Elbe Valley | 2.89, 0.19 | 2.38, 0.21 | 2, 0.30 | 4.05, 0.31 | 4, 0.29 | 4.4, 0.34 |
|  | The North Sea in Moonlight | 2.81, 0.24 | 2.34, 0.22 | 3.1, 0.30 | 3.35, 0.38 | 2.4, 0.38 | 2.95, 0.39 |
|  | The Waterfalls at Terni | 3.06, 0.21 | 3.30, 0.22 | 2.45, 0.35 | 3.9, 0.3 | 4.15, 0.25 | 3.95, 0.39 |
| NS | Aurore, novembre | 2.77, 0.24 | 2.05, 0.18 | 2.05, 0.25 | 3.7, 0.3 | 3.85, 0.31 | 4.25, 0.26 |
|  | Fontainebleau: Oak Trees at Bas-BrŽau | 2.61, 0.23 | 1.94, 0.18 | 2.2, 0.33 | 2.95, 0.32 | 2.7, 0.25 | 3, 0.32 |
|  | Bohemian Landscape with Mount Milleschauer | 2.49, 0.20 | 1.84, 0.19 | 1.95, 0.25 | 3.95, 0.39 | 4.2, 0.32 | 4.65, 0.31 |
|  | The Watzmann | 2.81, 0.23 | 1.54, 0.17 | 2.15, 0.31 | 3.55, 0.39 | 3.25, 0.30 | 3.4, 0.41 |
|  | The Oaktree in the Snow | 2.31, 0.24 | 1.72, 0.20 | 2.8, 0.36 | 3.35, 0.30 | 2.1, 0.36 | 3.05, 0.29 |
|  | Rocky Crags at l'Estaque | 2.82, 0.24 | 2.09, 0.23 | 3.25, 0.38 | 3.45, 0.34 | 3.2, 0.35 | 3.15, 0.39 |
